# Readability of health research informed consent forms: case of the National Health Research Ethics Committee in Tanzania

**DOI:** 10.1186/s12910-025-01200-w

**Published:** 2025-04-22

**Authors:** Renatha Kato, Renatha Joseph, Lazaro Haule, Mwanaidi Kafuye

**Affiliations:** 1 National Institute for Medical Research, 3 Barack Obama Drive, P.O. Box 9653, Dar es Salaam, 11101 Tanzania; 2 Department of Bioethics and Health Professionalism, School of Public Health and Social Sciences, Muhimbili University of Health and Allied Sciences, P.O. Box 65001, Dar es Salaam, Tanzania

**Keywords:** Informed consent form, Health, Readability, Research

## Abstract

**Background:**

Obtaining informed consent is the practice of respect for persons that gives the right to participants to make autonomous decisions about research participation. The difficult-to-read research informed consent forms (RICFs) hinder comprehension and can expose participants to harm. This study aims to assess the readability of health RICFs for studies approved by the National Health Research Ethics Committee (NatHREC) in Tanzania.

**Methods:**

We used a retrospective cross-sectional study design. A total of 266 RICFs were sampled from the NatHREC database using stratified and systematic random sampling strategies. The readability of RICFs was assessed using the Flesch Reading Ease (FRE) and Flesch-Kincaid Readability Grade Level (FKRGL) formulas available in Microsoft Word Office and by manual check. Data were collected using the assessment checklist, analyzed, and presented with SPSS and MS Excel software.

**Results:**

Out of 266 RICFs assessed, 65.4% had the recommended page numbers, 81.6% had longer sentences, and 80.5% were difficult to read, necessitating a person to acquire a US grade 10 (Form Four educational level in Tanzania) to understand the presented information. Pearson’s correlation coefficient with p-values of < 0.001 and 95% confidence level disclosed that sentence lengths in the RICFs had a statistical association with the difficult reading levels obtained.

**Conclusion:**

Findings from this study showed that most of the RICFs were concise in terms of page numbers and word count but had long and difficult sentences. Researchers should assess the readability of RICFs before submitting them for ethical approval. Research Ethics Committees (RECs) should consider inclusion of RICFs readability measurements in the Ethics Guidelines for Health Research. The study recommends further studies to assess the Kiswahili versions of RICFs to determine if the results obtained in this study apply to Kiswahili texts.

**Clinical trial number:**

Not applicable.

## Introduction

Obtaining informed consent is the practice of the principle of respect for persons that gives the right to potential research participants to make autonomous decisions on research participation. RICF is commonly used to convey research information to the participants [1–4]. It is a good practice to obtain written consent for any significant procedure when the person participates in a research project [5]. The consent is informed only if the participants have comprehended the study information [2]. The difficult-to-read RICFs can lead to poor comprehension of study information and can further cause physical and psychological harm to the participants.

RICFs have other issues such as increased length, more legalistic, and with a lot of technical terms which also affect comprehension. Some terminology can’t even be properly translated into local languages. Reduced understanding affects the participants’ choices about their involvement in research, threatens their safety, and may lead individuals to withdraw from enrollment, decline to take part in research, or they may consent to participate solely in hopes of resolving their healthcare issues [6–12]. Ethics Guidelines and some studies recommend the use of RICFs with readability scores from 60 to 70, reading grade levels less than or equal to a US grade 8, length of less than or equal to 3 pages, and less than 15 words per sentence for good disclosure and easier understanding [4, 9–13].

Studies conducted in Iran, the USA, Ireland, the UK, Norway, and China reported that more than half of the RICFs were difficult to read and had poor disclosure of study information. Studies in South Africa, Jordan, and Sudan showed that 2/3 of the RICFs were incomplete and difficult to read regardless of the language used [7, 9, 14]. Other studies conducted in Kenya and Uganda proved that more than 60% of RICFs were difficult to read; hence, participants had to rely on healthcare providers’/guardians’ advice [15–17].

Studies in Tanzania reported that not all potential research participants who attended information sessions understood the research information [18–20]. However, there is a lack of evidence on whether health RICFs used in Tanzania are easy to read and comprehend. Therefore, this study aimed to assess the readability of health RICFs for studies approved by the National Health Research Ethics Committee (NatHREC) in Tanzania.

## Methodology

### Study design

This was a retrospective cross-sectional study with a quantitative approach. This design was chosen because it is suitable when dealing with numerical data of the past to examine associations between variables [9, 21, 22]. We employed the quantitative approach in this study because the readability assessment was based on the reading grade levels, number of words, pages, and words per sentence in the RICFs.

### Study setting

This study was conducted at NatHREC, which is hosted at the NIMR Headquarters in the Dar es Salaam Region of Tanzania. NatHREC was picked because it is the national REC that reviews protocols from all over the country, including clinical trials, studies involving foreign research collaborators, and students whose institutions have no Institutional Review Boards (IRBs). Hence, NatHREC reviews many protocols along with their RICFs as compared to individual IRBs in Tanzania. Conducting the study at NatHREC helped us to have a large sample size that helped to draw a good conclusion.

### Study population

The target population for this study was the English versions of health RICFs for studies approved by NatHREC from 2020 to 2021. We chose the time interval from 2020 to 2021 because the Research Ethics Information Management System (REIMS) that bears the NatHREC online research databases became fully operational in early 2020. For that reason, the electronic copies of RICFs available in the NatHREC database are for research studies conducted from 2020 up to date.

### Sample size

The sample size for this study was 266 health RICFs which included 34 RICFs for clinical trial studies and 232 RICFs for non-clinical trial studies.

### Sample selection

We employed the stratified random sampling method in the sampling of RICFs from the NatHREC database to prevent the overrepresentation of non-clinical trials over clinical trial studies. Also, the systematic random sampling method was used to sample RICFs in each stratum at the interval of 3.

### Data collection methods and procedures

This study used the document review method to collect data from the RICFs. The data collection tool was the assessment checklist adapted from the reviewed literature [4, 7, 9, 14, 23]. RICFs in PDF format were converted to Word document format. Converted documents were verified for accuracy and any discrepancies were corrected. All information that could identify the investigators or study institutions (names and logos) was removed from the RICFs as was done in a readability study conducted in Kenya [15]. The researcher reviewed the RICFs to determine the types of research studies for which the forms were obtained. The RICFs were then classified based on the study types (Clinical and Non-clinical trials).

The Flesch Reading Ease (FRE) and Flesch-Kincaid Readability Grade Level (FKRGL) formulas available in the Microsoft Word Office were used to determine the readability scores, reading grade levels, number of sentences, words, and sentence lengths through the following steps: Step 1: The RICF document was opened in Word. Step 2: At the top toolbar, the “File” option was clicked, then “Options”. Step 3: The “Proofing” option was selected. Step 4: The “Show readability statistics” box was ticked. Step 5: The “OK” option was selected. Step 6: The “Spelling & Grammar” checkbox located at the top toolbar was clicked. Step 7: A small box appeared to the right of the RICF, showing options for correction of spelling and grammar. All suggested options were ignored. Step 8: Another small window popped up, showing the “Readability Statistics” of the opened RICF. Step 9: The readability statistics, including FRE scores, FKRGL scores, word count, and sentence lengths, were recorded in the checklist. Manual counting was used to obtain the page numbers for each RICF. The data from the assessment checklists was entered and kept on a computer secured with a password, accessible solely to the research investigators. The primary author kept the physical copies of the assessment checklists in a secured cabinet and will dispose of them following NatHREC guidelines.

### Data analysis

The principal investigator, who is also the primary author of this manuscript, carried out all tasks associated with data analysis.

### Readability scores of the research informed consent forms

The readability scores were grouped as “0 - <60 (Easy-to-read)”, “60–70 (Standard),” and “<70–100 (Hard-to-read)” and coded as “0”, “1,” and “2,” respectively. A standard readability score reflects how easy the content of the RICFs is to comprehend. While a score of 60 or higher, equivalent to a US reading grade level 8, is generally a good target, the standard readability can vary based on the intended audience [8, 24]. Conversely, a hard-to-read readability score indicates that the content of the RICF is not easy to read and understand because of several reasons, including unfamiliar words, length of sentences, paragraphs and words, and abbreviations. FRE scores below 60 are considered difficult to read, which further indicates that an average audience, particularly those with low health literacy, may need help to understand the content of the presented text [9, 14, 24, 25]. The coded data were entered in the IBM SPSS Statistics for Windows (Version 23.0) and analyzed to generate frequencies, median, maximum, and minimum scores. The percentages for each group were calculated. The results were presented by using a table.

### Reading grade levels of the research informed consent forms

The reading grade levels were grouped as “0 to ≤ 8 (Easy-to-read)” and “>8 (Hard-to-read)” and coded as “0” and “1” respectively. The coded data were entered in the IBM SPSS Statistics for Windows (Version 23.0) and analyzed to generate frequencies, median, maximum, and minimum reading grade levels. The percentages for each group were calculated. The results were presented by using a bar chart drawn using Microsoft Excel.

### Lengths of the research informed consent forms

The page numbers were grouped as “1–3 (standard)” and “>3 (long)” and coded as “0” and “1” respectively. Sentence lengths (words per sentence) for each RICF were grouped as “0 to < 15 (standard)” and “>15 (long)” and then as “0” and “1” respectively. The number of sentences and words were not grouped. The coded data for page numbers and sentence lengths were entered in the IBM SPSS Statistics for Windows (Version 23.0) and analyzed to generate frequencies, median, maximum, and minimum scores.

Percentages of scores for sentence length were calculated. The number of sentences and words in the RICFs were entered in the IBM SPSS software and were analyzed to generate median, maximum, and minimum scores. The RICFs’ page numbers were presented by using a bar chart drawn using Microsoft Excel while the number of words and sentence lengths were presented by using tables. A Pearson’s correlation test was performed to find the association between the study variables. The correlation coefficient with a p-value of < 0.005 and 95% confidence level were regarded as statistically significant.

### Ethical consideration

This study obtained the Ethics Clearance Certificate number MUHAS-REC-02-2023-1641 dated 24/04/2023 from the Muhimbili University of Health and Allied Sciences Institutional Review Board (MUHAS IRB) including a waiver of the consent process. We also obtained permission from NIMR to use the electronic copies of English versions RICFs for studies approved by NatHREC from 2020 to 2021. To ensure confidentiality, we removed all information that might identify the investigators or study institutions from the RICFs. RICFs were identified by using the ID numbers.

## Results

### Characteristics of the research informed consent forms

This study assessed 266 RICFs for approved clinical and non-clinical trial studies. Among the assessed RICFs, 129 (48.5%) were approved in 2020, while 137 (51.5%) were approved in 2021. Out of all RICFs, 34 (12.8%) were for clinical trial studies, while 232 (87.2%) were for non-clinical trials.

### Readability scores of the research informed consent forms

The median FRE score of all RICFs was 53.1. The FRE scores > 70–100 (easy readability) were 4(1.5%), 60–70 (standard readability) were 48(18%), and < 60 (hard to read) were 214 (80.5%). The maximum FRE score was 75.8, while the minimum score was 26.3. RICFs for both clinical and non-clinical trials had a median FRE score of less than 60 (Table 1).

**Table 1:** Flesch reading ease scores of the assessed RICFs

FRE scores	Clinical trials	Non-clinical trials	All studies
	*n*(%)	*n*(%)	*n*(%)
>70 (Easy-to-read)	2 (5.9)	2 (0.9)	4 (1.5)
60 - 70 (Standard)	9 (26.5)	39 (16.8)	48 (18)
< 60 (Hard-to-read)	23 (67.6)	191 (82.3)	214 (80.5)
Median	59.1	52.6	53.1
Maximum	75.8	71.7	75.8
Minimum	45.3	26.3	26.3

### Reading grade levels of the research informed consent forms

**Fig. 1:**
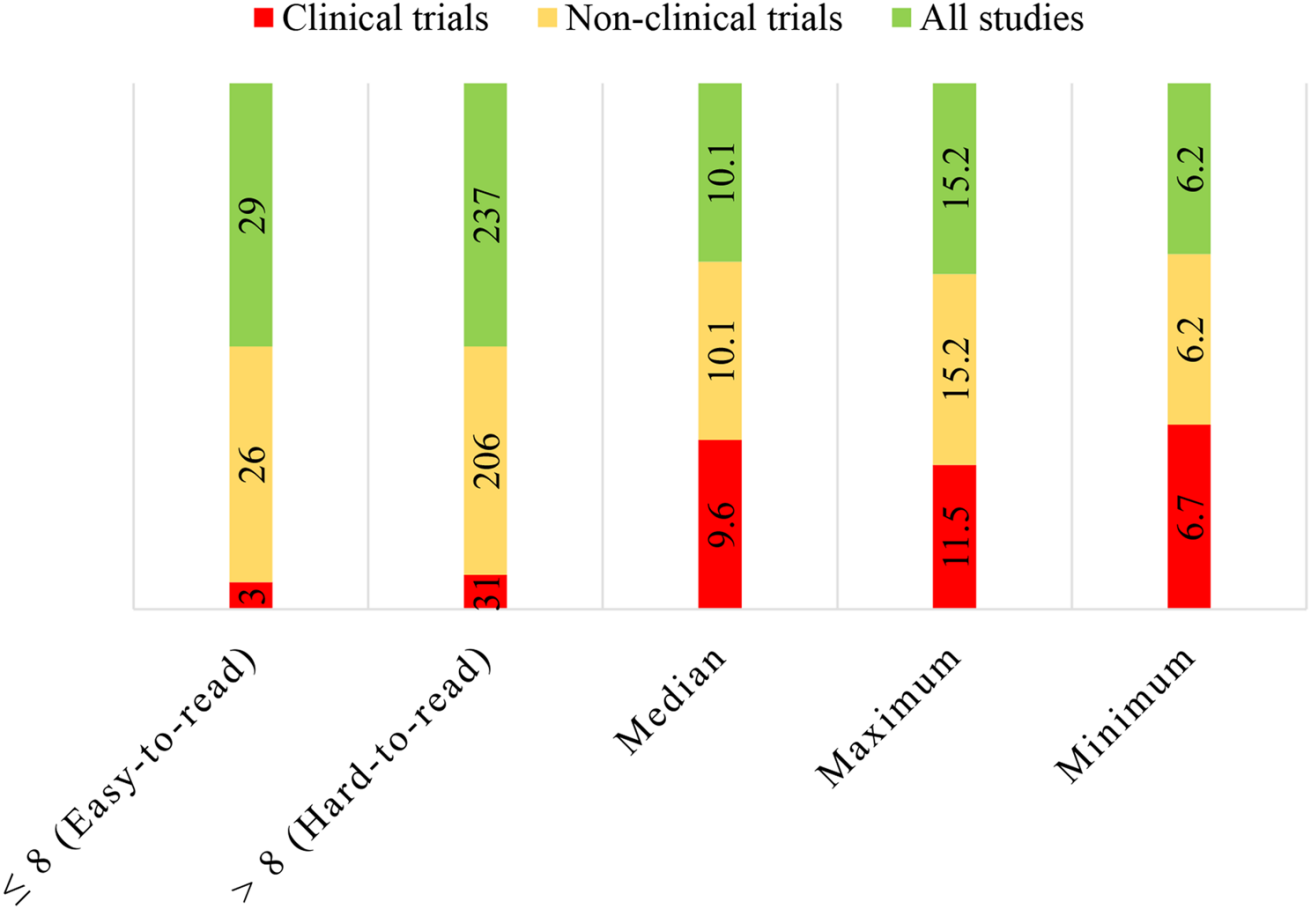
Flesch-Kincaid reading grade levels of the assessed RICFs

The median Flesch-Kincaid Reading Grade Level of all RICFs was 10.1. The RICFs that had reading grade levels less than or equal to 8 (easy-to-read) were 29(10.9%), while 237(89.1%) had grade levels above 8 (hard-to-read). The maximum reading grade level was 15.2, and the minimum was 6.2. The clinical trials RICFs that had easy-to-read grade levels were 3(8.8%), whereas 31(91.2%) had the hard-to-read grade levels. Non-clinical trials RICFs that had easy-to-read grade levels were 26(11.2%) while 206(88.8%) had difficult-to-read grade levels (Fig. 1).

### Lengths of the research informed consent forms

#### Page lengths

**Fig. 2:**
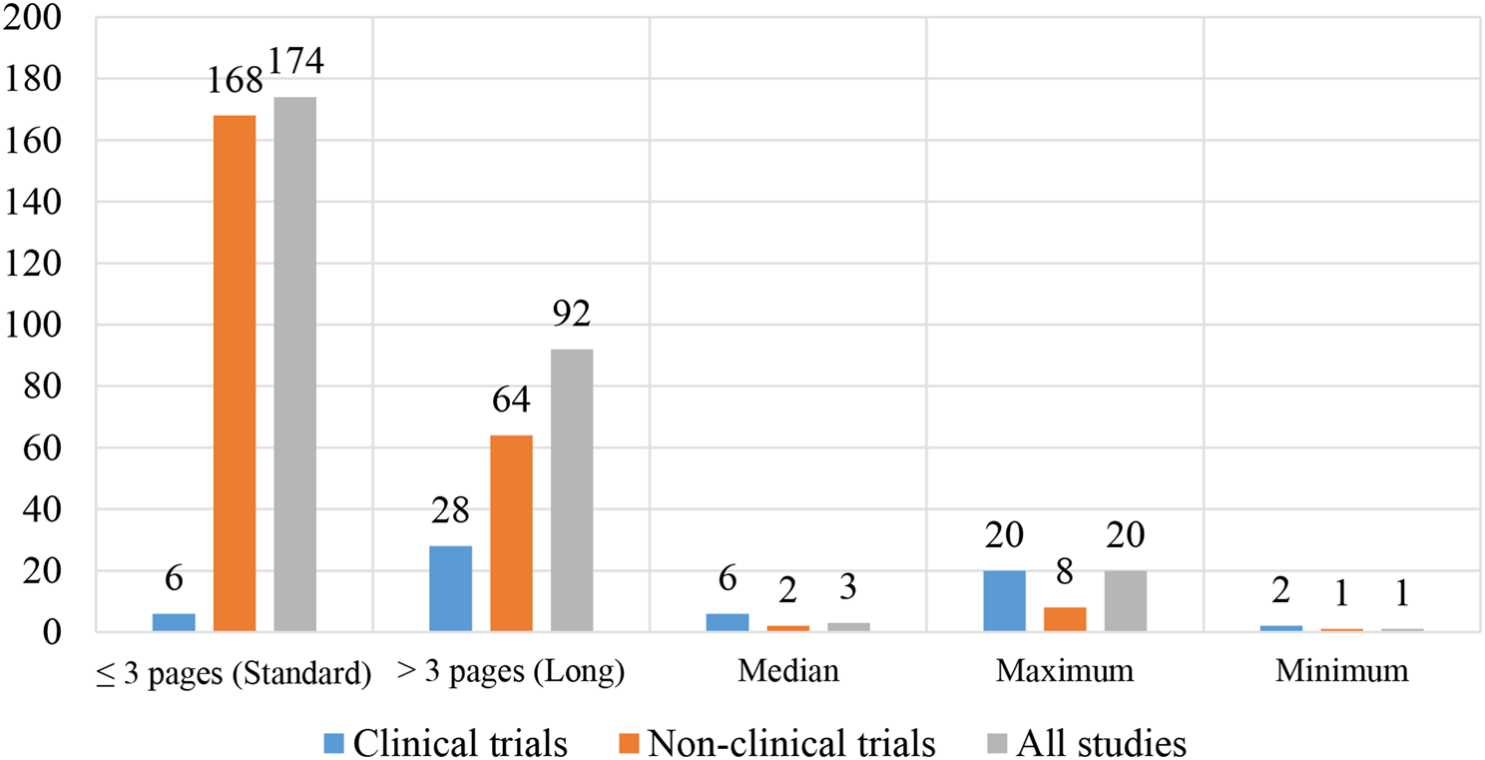
Page lengths of the assessed RICFs

Out of all RICFs assessed, 174(65.4%) had less than or equal to 3 pages, and 92(34.6%) had more than 3 pages. The median page length for all RICFs was 3 pages. The maximum page length of all RICFs assessed was 20 pages, while the minimum length was 1 page. The median page length for clinical trials RICFs was 6 pages, and for non-clinical trials RICFs was 2 pages (Fig. 2).

### Word count of the research informed consent forms

The median word count for all RICFs was 832 words. The maximum word count per RICF was 7021 words while the minimum number was 130 words. The median word count for clinical trials RICFs was 2246 words while for non-clinical trials RICFs was 769.5 words (Table 2).

**Table 2:** Word count of the assessed RICFs

Word count	Clinical trials	Non-clinical trials	All studies
Median	2246	769.5	832
Maximum	7021	3346	7021
Minimum	731	130	130

### Sentence length of the research informed consent forms

The median sentence length for all RICFs was 17.4 words per sentence. The RICFs that had less than 15 words per sentence (standard sentence length) were 49(18.4%) while 217(81.6%) had more than 15 words per sentence (long sentences). The maximum number of words per sentence was 26.5 while the minimum number was 8.2 words per sentence (Table 3).

**Table 3:** Number of words per sentence

Words per sentence	Clinical trials *n*(%)	Non-clinical trials *n*(%)	All studies *n*(%)
<15 words (Standard)	1(2.9)	48(20.7)	49(18.4)
>15 word (Long)	33(97.1)	184(79.3)	217(81.6)
Median	18.6	17.3	17.4
Maximum	21.5	26.5	26.5
Minimum	15.1	8.2	8.2

### Correlation and significance between readability variables of the research informed consent forms

A Pearson’s correlation coefficient with a p-value of < 0.05 and 95% confidence level disclosed that the length of sentences in the RICFs had a statistical association with the reading level scores (Table 4).

**Table 4:** Correlation between FRE scores, reading levels, and lengths of the RICFs

Variables	FRE Score	FKRG Level	Pages	Words	Words per sentence
**FRE Score**					
Correlation	1	-0.922	0.224	0.250	-0.347
p (2-tailed)		0.000	0.000	0.000	0.000
**FKRG Level**					
Correlation	-0.922	1	-0.111	-0.113	0.670
p (2-tailed)	0.000		0.071	0.066	0.000

## Discussion

### RICFs’ readability scores

Findings from this study indicated that more than half of the RICFs had less than 60 FRE scores expressing their difficulty. A difficult readability can affect comprehension and, hence, can prevent potential participants from providing informed consent [26]. These findings are in line with two studies conducted in South Africa, which reported that most of the studied RICFs (53% and 66.7% respectively) were difficult to read [9, 10]. These results are also similar to that of a study in France, which indicated that the RICFs’ readability scores were hard to read regardless of the diversity of protocols included [27]. The hard-to-read RICFs are one of the causes of poor comprehension by participants, hence affecting their voluntariness to participate in the study [28]. This is critical in clinical trial studies and may pose a potential threat to the participants’ welfare and safety, especially when new interventions (drugs or devices) are being tested on participants [29]. The low readability scores identified in this study could serve to alert the REC’s reviewers to consider this aspect of RICFs that is not so obvious.

RECs reviewers can ensure RICFs are readable by first advising researchers to conduct pilot tests of their RICFs to identify confusing parts. Second, employing the standardized readability formulas available in Microsoft Word Office to confirm whether the reading levels of the submitted RICFs meet the standards prior to approval. FRE score greater than or equal to 80 (equivalent to Grade Seven in Tanzania) is suitable for RICFs used in Tanzania. This is because the majority of Tanzanians have attained primary school education. According to the World Bank and UNESCO Institute of Statistics, in 2020 the primary school completion rate in Tanzania was 66% for boys and 72% for girls, as compared to the secondary school rate of 32% for men and 35% for women, and tertiary education rate of 9% for men and 7% for women [30].

Lastly, the REC reviewers should routinely examine the readability metrics in submitted RICFs as part of the approval process to verify that the key aspects that reduce reading difficulties have been addressed. This includes ensuring that the RICFs define complex words using simpler terms, avoid using medical jargons or explain them if they must be used, refrain from using less familiar vocabulary, exclude non-essential details, utilize the second person instead of the first or third person, prefer active voice over passive voice, aim for concise sentences (preferably less than 20 words) and paragraphs or use lists when possible (less than 10 lines), maintain a reasonable length (preferably 2–3 pages for non-clinical trials and 5 to 6 pages for clinical trials), use large font sizes (preferably size 12), select an easy-to-read font style (preferably Times New Roman), ensure no overcrowding of text, provide sufficient spacing (line spacing of 1.15, which is the default in Microsoft Word), and include clear headings and subheadings [14, 31–33].

### Reading grade levels of the research informed consent forms

The results from this study showed that more than half of the RICFs had difficulty reading levels. The obtained median of 10.1 necessitates a person to acquire a minimum of grade 10 (equivalent to a Form Four educational level in Tanzania) to be able to understand the presented information. These results were consistent with studies conducted by Fischer in South Africa and Elaine in the USA, which reported that the average reading levels for all RICFs studied were equivalent to a US grade 10 [9, 40]. Although the Tanzania health dataset lacks health literacy measurements, reading grade level 10 is difficult-to-read and comprehend for most Tanzanians. This is proved by a study conducted in the Morogoro region of Tanzania, which showed that 67.1% of the respondents had marginal and inadequate health literacy [34].

Reading level 10 exceeds the readability standards recommended by various countries and institutions. For example, the United Kingdom (UK) Government recommends the use of documents written on reading grade level 5 [35], and the American Medical Association (AMA) and the National Institutes of Health (NIH) recommend a reading level less than or equal to grade 6 for easy comprehension of health information [36]. The University of Cape Town’s Human Research Ethics Committee (UCTHREC) in South Africa requires that language in the ICFs be at a reading level equivalent to that of grades 6 to 8 educational reading level [10]. Tanzania’s national guidelines are yet to emphasize readability and reading levels of health RICFs [13], which leaves room for the REC to update them to include concrete guidelines for sentence length, word choice, and document length.

The CIOMS (2016) and several readability studies emphasize that participants’ reading ability and education level are important factors in making them understand the written information to allow informed decisions [4, 8, 37, 38]. Simple and readable RICFs can remove barriers in the recruitment of participants with lower reading comprehension levels, creating a more complete sample while also ensuring that participants are aware of the potential benefits and risks of participation [9].

Findings in this study indicate that participants with low health literacy were not considered during the preparation of informed consent forms. This further implies that the participants had difficulty understanding the research information before they agreed to participate in the studies. Hence, the informed consent was not clearly obtained.

### Lengths of the research informed consent forms

Most of the RICFs met the page lengths recommended by the CIOMS 2016 of not more than 3 pages per RICF [4]. These results show that, in most RICFs, the page numbers were not a barrier to comprehension of study information. The median word count was 832 words. Based on the reading speed of America (240 words per minute), the results in this study indicate that it could take less than 5 min for an average reader to read a complete RICF [39]. However, in African countries including Tanzania, it could take more time than that because most participants have low literacy. The results from this study are different from those of studies conducted in South Africa and America which found that RICFs had a median of 5 pages and a mean of 10.3 pages respectively [9, 40]. The page lengths and word count in this study show that it was easy for a participant to read a complete RICF.

The median number of words per sentence was 17.4 showing that most of the RICFs had longer sentences (> 15 words per sentence) than what has been recommended by different studies. Long sentences are among the factors that affect the readability standards of a document. The use of short sentences helps to simplify language and eventually ensures comprehension of the research information [9, 14, 32]. A study conducted in Ghana highlighted that sentence length is an essential indicator of reading difficulty [25]. Therefore, the difficult readability obtained in this study is associated with long sentences used in the RICFs.

A clear distinction was observed between clinical and non-clinical trials in the number of pages and words. Clinical trials ICFs had many pages and words and had longer sentences than the non-clinical trials ICFs. As a result, only 8.8% of clinical trials RICFs had easy reading levels as compared to 11.2% of non-clinical trials RICFs that had acceptable reading levels. The increased length of clinical trials ICFs might be because they had longer descriptions of the randomization process and follow-up visits under the study procedures section. Complicated medical procedures increase the lengths of the RICFs [41]. These results conform with a study conducted in Norway which reported that clinical trials ICFs include a lot of information about the participant’s diagnosis and treatment, which increases length of the RICFs [42].

The results from this study pose the need to reduce sentence lengths in RICFs for all studies and to reduce the word count and page numbers in clinical trials. By doing so, it will be easy for participants to read and understand the study information presented in the RICFs. Generally, the results from this study imply that the RICFs were concise in terms of page numbers and word count but had long sentences that were tedious to read. This might have led to blind informed consent.

### Future revision of RICFs and training researchers on the readability of RICFs

We plan to conduct training sessions for researchers aimed at raising awareness of RICF readability and its assessment. This initiative will be more successful with the participation of various stakeholders, especially language specialists in English and Kiswahili, along with local RECs. If possible, we will also involve experts in indigenous languages to assist communities that may not comprehend Kiswahili or English, as this language barrier could increase their vulnerabilities. Furthermore, we will look for possible organizations to facilitate this vital initiative.

### Study strengths, limitations, and mitigation measures

This is the first study for the assessment of readability of the health RICFs in Tanzania. Results from this study pave the way for future research and the inclusion of measurements of the RICFs’ readability in the ethics guidelines. This study had some limitations. Firstly, we obtained the RICFs from a single research site, which minimizes variability. To mitigate this limitation, we selected the national REC that has more approved RICFs than IRBs to have enough sample size. Secondly, the readability formulas used in this study were unable to assess readability factors like page numbers. We mitigated this limitation by conducting a manual count of the RICFs’ page numbers.

Lastly, the assessment was limited to the English language RICFs due to a lack of software for the assessment of the Kiswahili language RICFs. Although this study only examines English-language RICFs, various studies indicate that RICFs translated to local languages are not simple or easy to read. Studies in Kenya, India, and South Africa reported that most of the local language RICFs and patient information leaflets (Kiswahili, Hindu, isiXhosa, and Afrikaans) were consistently very difficult to read as compared to the same documents in the English language. In South Africa, this change was associated with the fact that the Afrikaans languages often use more words to express the same idea that could be expressed in fewer words in the English language[10, 15, 43, 44]. This applies to the Kiswahili language. Hence, we recommend further studies to assess and confirm if the results obtained in this study apply to Kiswahili language RICFs submitted to NatHREC.

## Conclusion

Findings from this study showed that most of the RICFs were concise in terms of page numbers and word count but had long and difficult sentences. Research investigators should assess the readability of the RICFs before submitting them for ethical approval. RECs should consider the inclusion of readability measurements of RICFs in the Ethics Guidelines for Health Research. Additionally, further studies should be conducted to assess the Kiswahili versions of RICFs to determine if the results obtained in this study apply to Kiswahili texts.

## Data Availability

Data supporting the results reported in this article are available from the corresponding author upon reasonable request.

## Abbreviations

AMA: American Medical Association
CIOMS: Council for International Organizations of Medical Sciences
FKRGL: Flesch- Kincaid Readability Grade Level
FRE: Flesch Reading Ease
IBM: International Business Machines Corporation
IRB: Institutional Review Board
MUHAS: Muhimbili University of Health and Allied Sciences
NatHREC: National Health Research Ethics Committee
NIH: National Institutes of Health
NIMR: National Institute for Medical Research
REC: Research Ethics Committee
REIMS: Research Ethics Information Management System
RICF: Research Informed Consent Form
SPSS: Statistical Package for the Social Sciences
UCTHREC: University of Cape Town’s Human Research Ethics Committee
